# Supplementary material for: Thiacalixarenes with Sulfur Functionalities at Lower Rim: Heavy Metal Ion Binding in Solution and 2D-Confined Space
Source: Int J Mol Sci. 2022 Feb 20;23(4):2341. doi: 10.3390/ijms23042341 (PMC8875454; doi:10.3390/ijms23042341)
Supplement: Supplementary file 1 [file ijms-23-02341-s001.zip › ijms-1558199-supplementary.pdf]

Thiacalixarenes with sulfur functionalities at lower rim: heavy metal ion binding in solution and 2D-confined space

Anton Muravev[1,*], Ayrat Yakupov[2], Tatiana Gerasimova[1], Daut Islamov[1], Vladimir Lazarenko[3], Alexander Shokurov[4], Alexander Ovsyannikov[1], Pavel Dorovatovskii[3], Yan Zubavichus[5], Alexander Naumkin[4,6], Sofiya Selektor[4], Svetlana Solovieva[1], and Igor Antipin[2]

[1]Arbuzov Institute of Organic and Physical Chemistry, FRC Kazan Scientific Center, Russian Academy of Sciences, Arbuzov str. 8, 420088 Kazan, Russia

[2]Butlerov Institute of Chemistry, Kazan Federal University, Kremlevskaya str. 18, 420008 Kazan, Russia

[3]National Research Centre "Kurchatov Institute", Kurchatov Square 1, 123182 Moscow, Russia

[4]Frumkin Institute of Physical Chemistry and Electrochemistry, Russian Academy of Sciences, Leninskii pr. 31, 119071 Moscow, Russia

[5]Synchrotron Radiation Facility SKIF, Boreskov Institute of Catalysis, Siberian Branch, Russian Academy of Sciences, Nikolskii pr. 1, 630559 Koltsovo, Russia

[6]Nesmeyanov Institute of Organoelement Compounds, Russian Academy of Sciences, Vavilov str. 28, 119334 Moscow, Russia

*E-mail: antonm@iopc.ru

Electronic Supplementary Information

Table of Contents:

DLS measurements	S2
Crystallographic data	S2
Spectrophotometric titration data	S3
Analysis of frontier molecular orbitals	S4
Langmuir monolayer measurements	S5
XPS spectra	S6
Copies of NMR spectra	S7

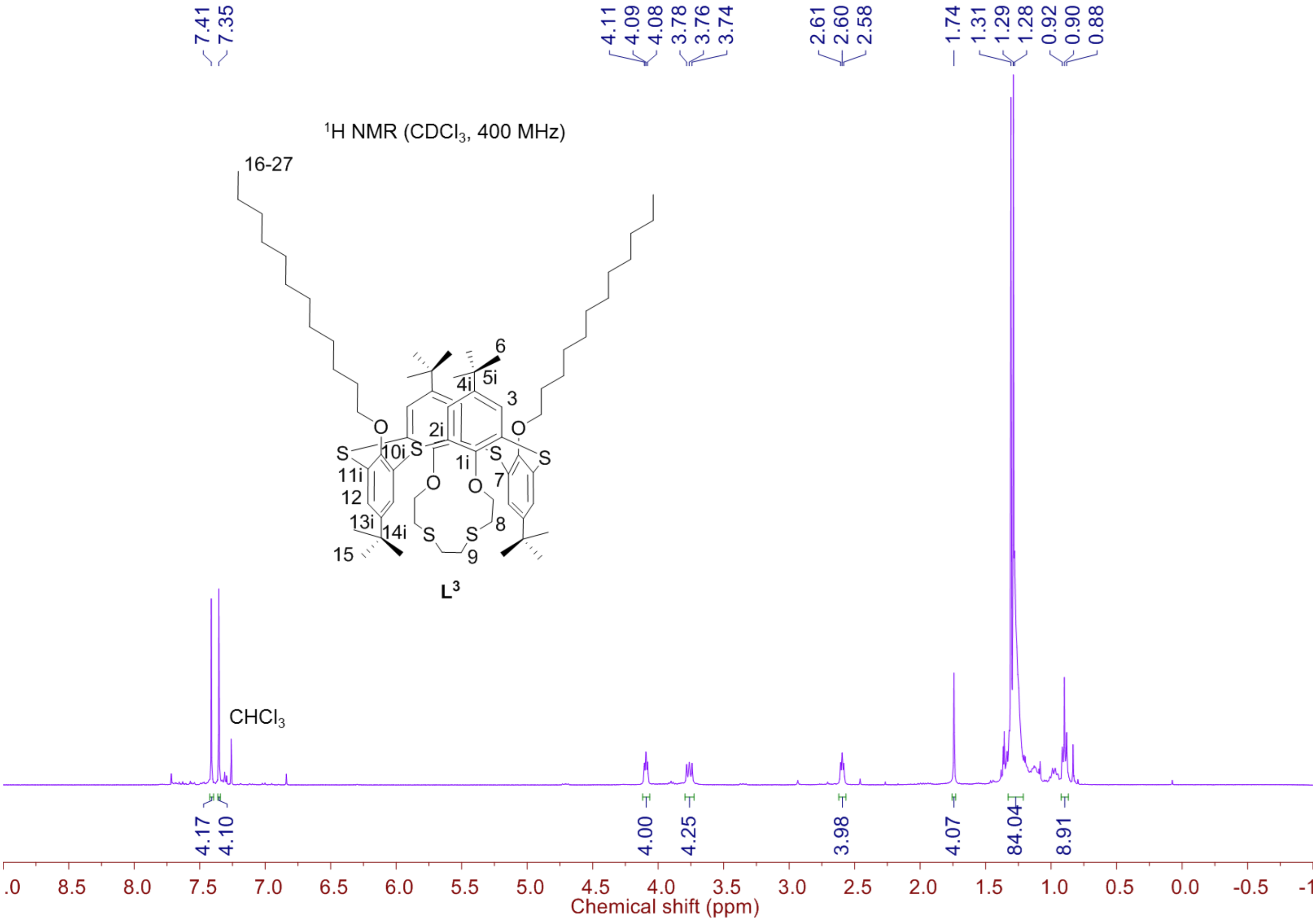

Figure S1. ^{1}H NMR spectrum of compound **L^3** ($CDCl_3$, 400 MHz, 303 K).

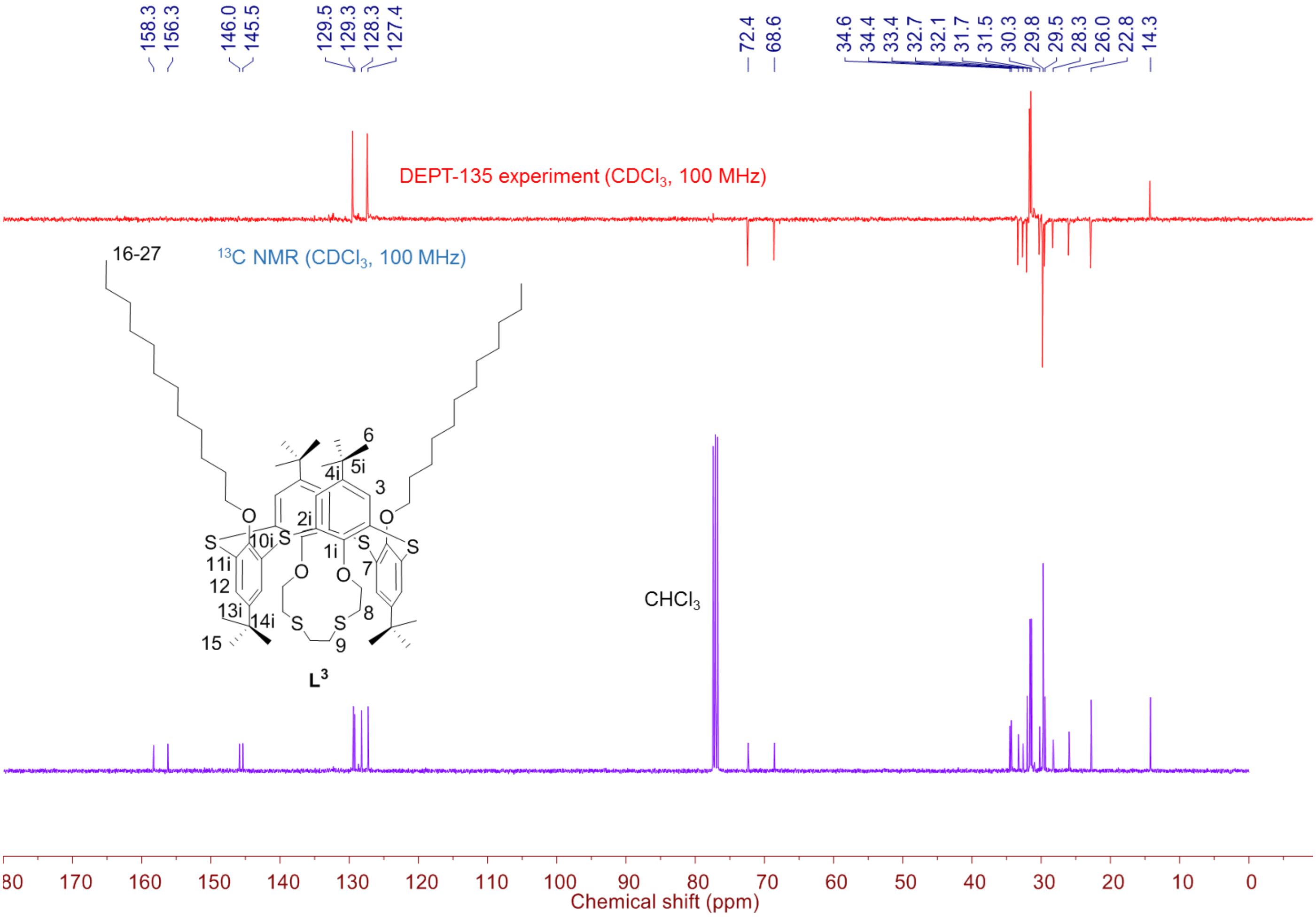

Figure S2. ^{13}C NMR spectrum and DEPT-135 experiment of compound **L^3** ($CDCl_3$, 100 MHz, 303 K).

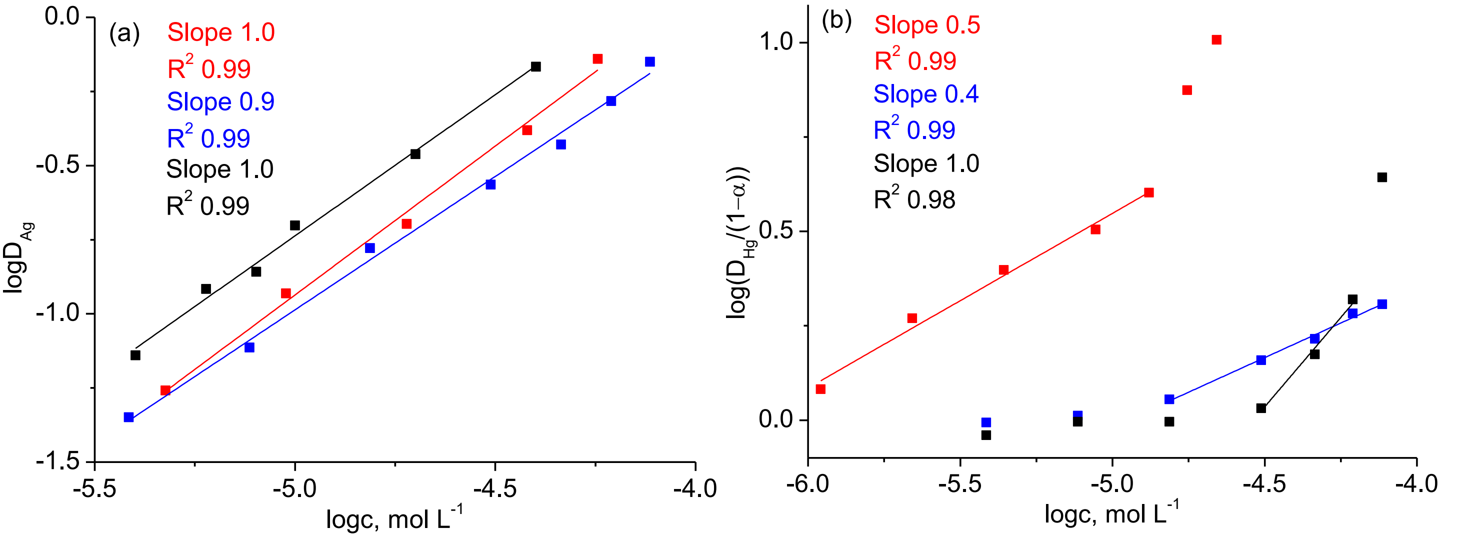

Figure S3. Plots of (a) logD_{Ag} and (b) log(D_{Hg}/(1–α)) against log*c* for thioethers **L**^{2} (red line), **L**^{3} (blue line), and **L**^{4} (black line) in the DCM–H_2O system (*c* (AgPic) = 1 × 10^{-4} *M* and *c* ($HgPic_2$) = 1 × 10^{-4} *M* (ligand **L**^{2}) or 5 × 10^{-5} *M* (ligands **L**^{3} and **L**^{4}).

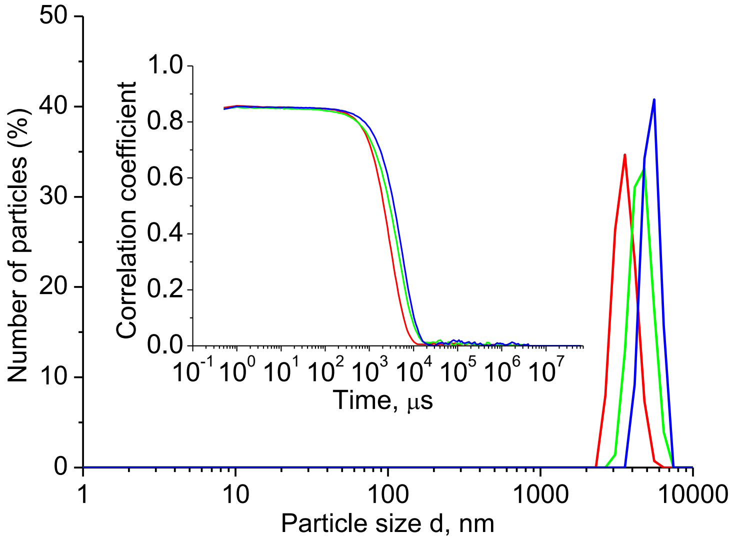

Figure S4. Number-averaged particle size distribution and corresponding correlograms of CH_2Cl_2 phase after mixing with aqueous phase containing $Hg(NO_3)_2$ dissolved in tris·HCl buffer in picric acid (blue curves) and CH_2Cl_2 phase with dissolved compound **L**^{2} after mixing with aqueous phase containing picric acid and tris·HCl buffer (red curve) and aqueous phase containing $Hg(NO_3)_2$ (green curve).

Table S1. Crystallographic parameters of the metal complexes

Formula	$[Ag_2$**L**$^2{}_2](ClO_4)_2$, $C_{92}H_{120}Ag_2O_8S_{12}$, $2(ClO_4)$	$[Ag_2$**L**$^2(ClO_4)_2]_n$, $C_{46}H_{60}Ag_2Cl_2O_{12}S_6$, $2(CHCl_3)$	$[Hg_2$**L**$^4Cl_4]$, $C_{92}H_{116}Cl_4Hg_2O_8S_{12}$
Molecular weight	2153.23	1522.67	2277.54
Crystal system	triclinic	orthorhombic	triclinic
Space group	*P*-1	*Pbca*	*P*-1
a(Å)	12.200(2)	18.075(4)	10.115(2)
b(Å)	15.200(3)	19.726(4)	15.338(3)
c(Å)	16.680(3))	35.121(7)	19.017(4)
α(deg)	76.01(3)	90	96.48(3)
β(deg)	77.06(3)	90	101.05(3)
γ(deg)	75.59(3)	90	99.55(3)
V(Å^3)	2862.5(12)	12522(4)	2823.4(11)
Z	1	8	1
Colour	Colourless	Colourless	Colourless
Crystal dim (mm^3)	0.03 x 0.02 x 0.02	0.03 x 0.02 x 0.02	0.03 x 0.02 x 0.02
D_{calc} (gcm^{-3})	1.249	1.615	1.339
F(000)	1120	6176	7180
μ (mm^{-1})	0.740	1.333	0.632
Wavelength (Å)	0.7450	0.7450	0.80246
Number of data measurements	48689	129153	44088

Number of data with I> 2σ(I)	15666 [R(int) = 0.0420]	17272 [R(int) = 0.0410]	12340 [R(int) = 0.1087]
R (%)	5.43	3.08	8.01
R_w (%)	15.84	8.41	22.27
GOF	1.042	1.078	1.036
Largest diff. peak and hole (eÅ^{-3})	1.380 and -1.683	1.700 and -1.382	2.424 and -2.555

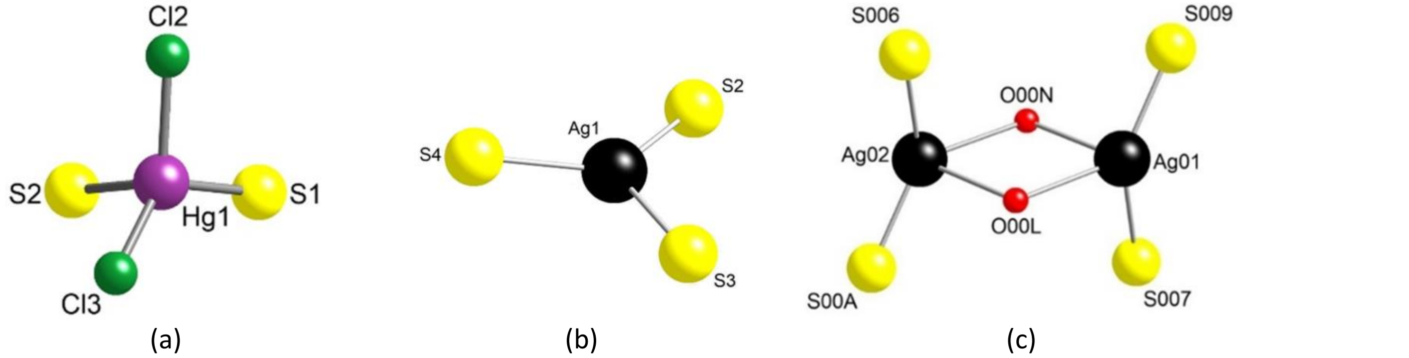

Figure S5. Coordination environment of metal ions in (a) [Hg_2**L**^{4}Cl_4], (b) [Ag_2**L**^{2}$_2$]$(ClO_4)_2$, and (c) [Ag_2**L**^{2}$(ClO_4)_2$]$_n$.

Table S2. Bond distances and angles for metal coordination environment in [Hg_2**L**^{4}Cl_4], [Ag_2**L**^{2}$_2$]$(ClO_4)_2$, and [Ag_2**L**^{2}$(ClO_4)_2$]$_n$

[Hg_2**L**^{4}Cl_4]	[Ag_2**L**^{2}$_2$]$(ClO_4)_2$	[Ag_2**L**^{2}$(ClO_4)_2$]$_n$	
d (Hg1–S1) = 2.579(8) Å	*d* (Ag1–S2) = 2.520(6) Å	*d* (Ag01–S007) = 2.586(7) Å	∠S007Ag01S009 = 135.38(6)°
d (Hg1–S2) = 2.789(5) Å	*d* (Ag1–S3) = 2.553(1) Å	*d* (Ag01–S009) = 2.472(1) Å	∠S007Ag01O00N = 91.32(0)°
d (Hg1–Cl2) = 2.397(8) Å	*d* (Ag1–S4) = 2.570(2) Å	*d* (Ag01–O00L) = 2.492(2) Å	∠S009Ag01O00L = 131.23(4)°
d (Hg1–Cl3) = 2.340(0) Å	∠S4Ag1S2 = 132.59(7)°	*d* (Ag01–O00N) = 2.681(8) Å	∠S006Ag02O00N = 90.72(4)°
∠Cl2Hg1S1 = 98.26(3)°	∠S2Ag1S3 = 115.00(0)°	*d* (Ag02–S00A) = 2.480(2) Å	∠S006Ag02O00L = 88.10(6)°
∠S1Hg1Cl3 = 127.51(2)°	∠S3Ag1S4 = 112.03(7)°	*d* (Ag02–S006) = 2.585(6) Å	∠O00NAg02O00L = 70.26(0)°
∠Cl3Hg1S2 = 101.22(6)°		*d* (Ag02–O00L) = 2.739(3) Å	∠O00LAg02S00A = 91.84(4)°
∠S2Hg1Cl2 = 102.03(8)°		*d* (Ag02–O00N) = 2.514(2) Å	∠S00AAg02S006 = 135.73(0)°
∠Cl2Hg1Cl3 = 130.54(5)°		∠S009Ag01O00N = 91.02(2)°	∠S00AAg02O00N = 130.58(7)°
∠S1Hg1S2 = 83.00(4)°		∠O00NAg01O00L = 71.55(0)°	∠Ag01O00NAg02 = 109.67(2)°
		∠O00LAg01S007 = 91.37(0)°	∠Ag01O00LAg02 = 108.51(9)°

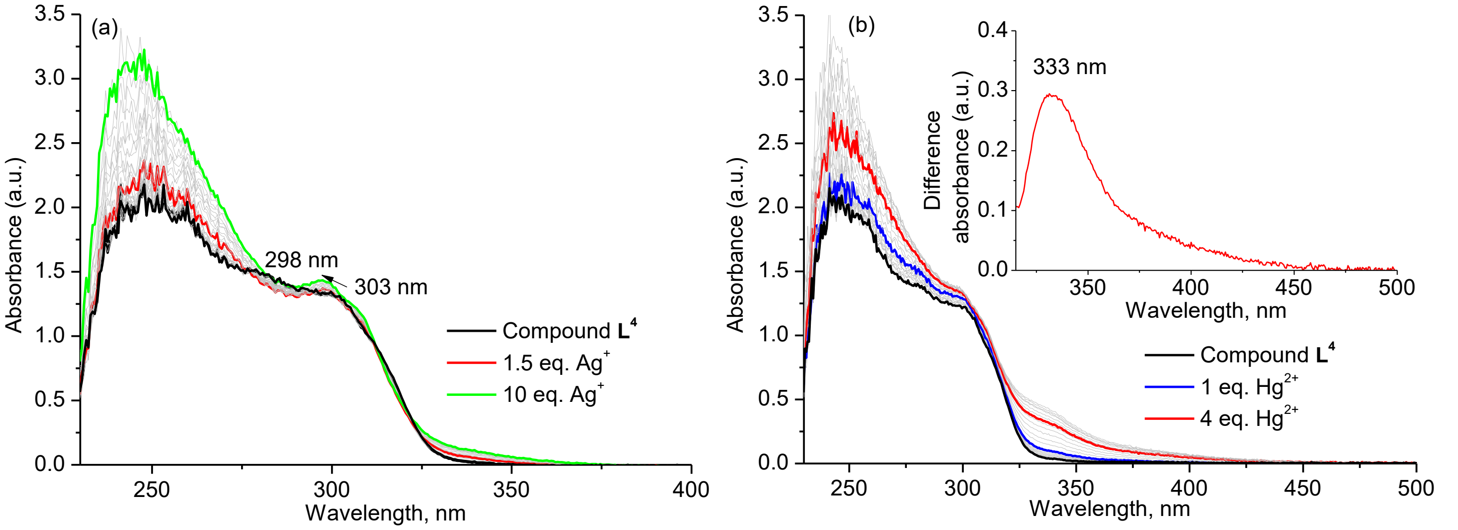

Figure S6. Evolution of UV/Vis absorption spectra of compound **L**^{4} ($c = 1 \times 10^{-5}$ *M* in 10:1 CH_2Cl_2–MeOH) on addition of (a) Ag^+ and (b) Hg^{2+} as perchlorates. Inset shows difference spectrum of ligand **L**^{4} and its Hg complex.

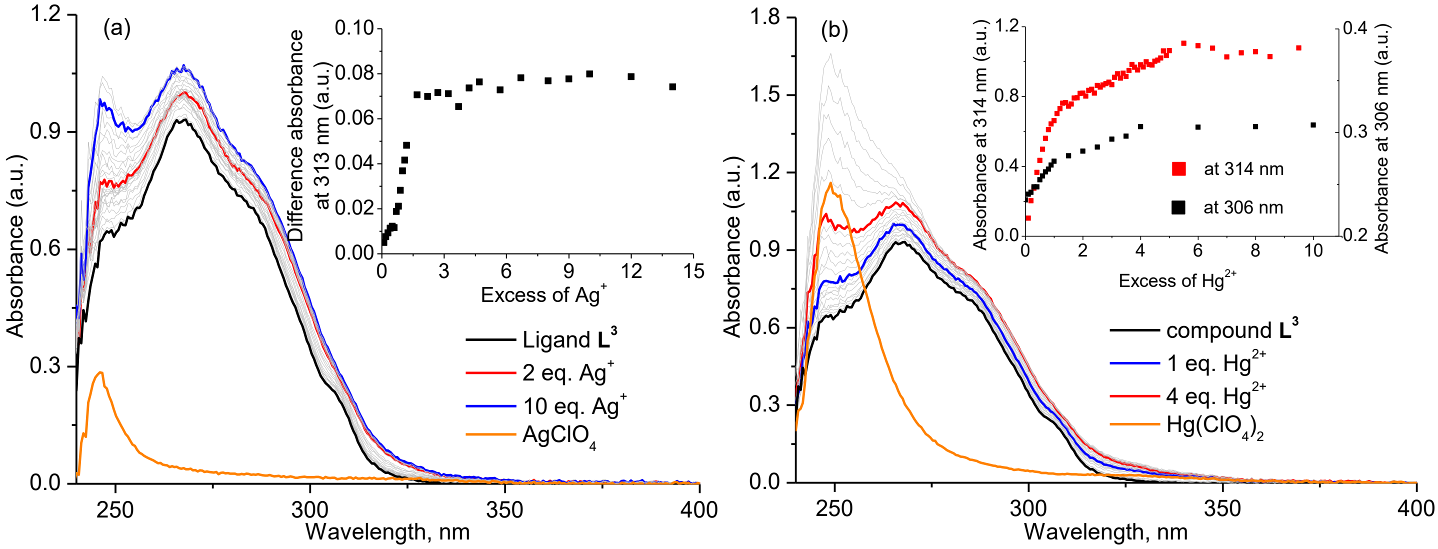

Figure S7. Evolution of UV/Vis absorption spectra of compound **L³** ($c = 1 \times 10^{-5}$ *M* in 10:1 CH_2Cl_2–MeOH) on addition of Ag^+ and Hg^{2+} as perchlorates. Insets show scatter plots of titration.

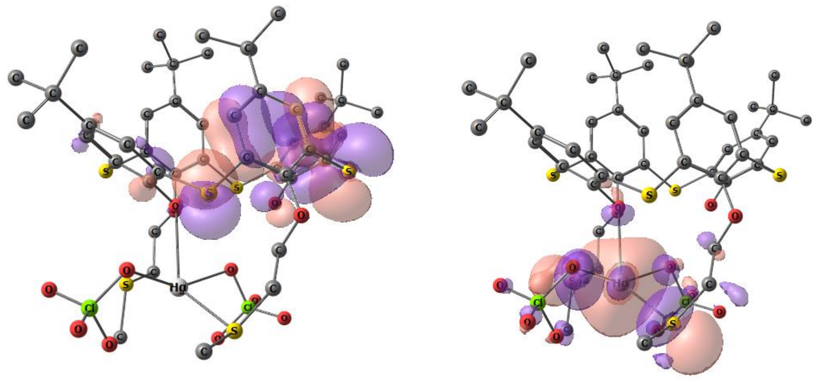

Figure S8. Frontier molecular orbitals contributing to low-energy absorption bands of **L²-Hg-a** complex: HOMO (left) and LUMO (right).

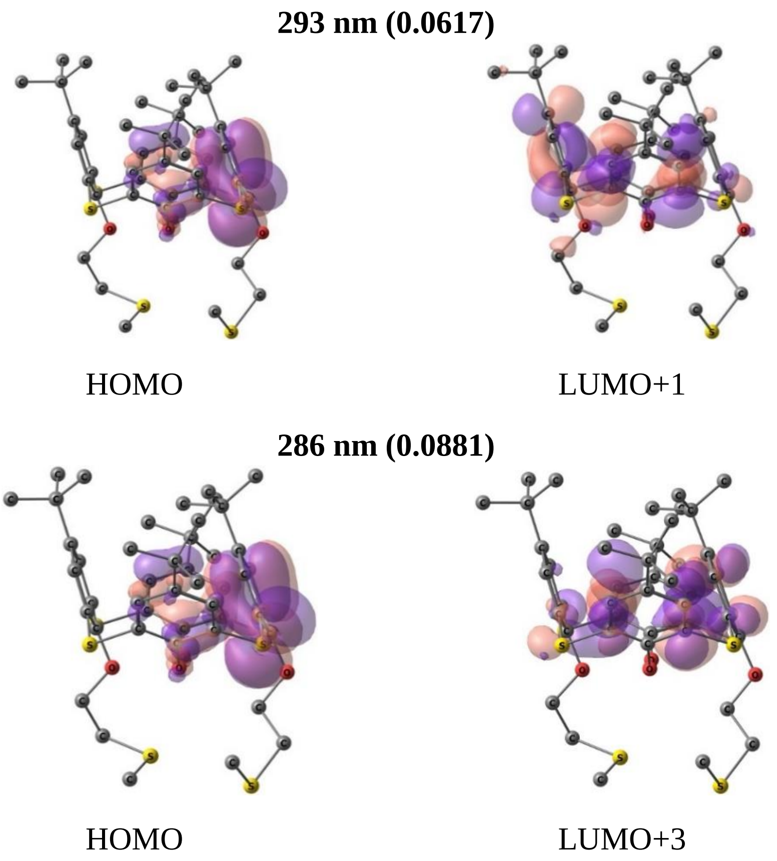

Figure S9. Frontier molecular orbitals contributing to low-energy absorption bands of ligand **L²**.

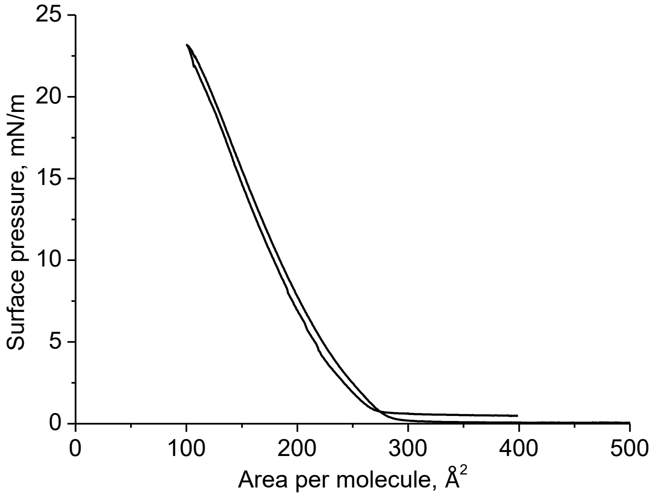

Figure S10. Monolayer compression–expansion cycle of compound **L⁴** at the air–water interface ($c = 5 \times 10^{-5}$ *M* in $CHCl_3$).

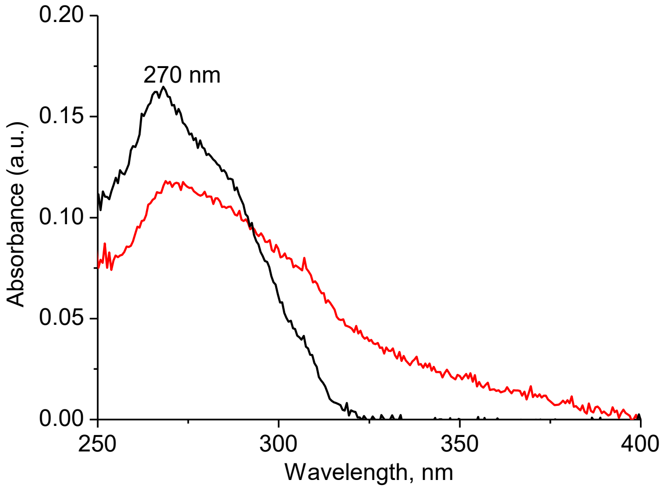

Figure S11. UV/Vis spectra of compound **L³** in $CHCl_3$ ($c = 2 \times 10^{-6}$ *M*) (black curve) and at air–water interface (π = 17 mN/m) (red curve).

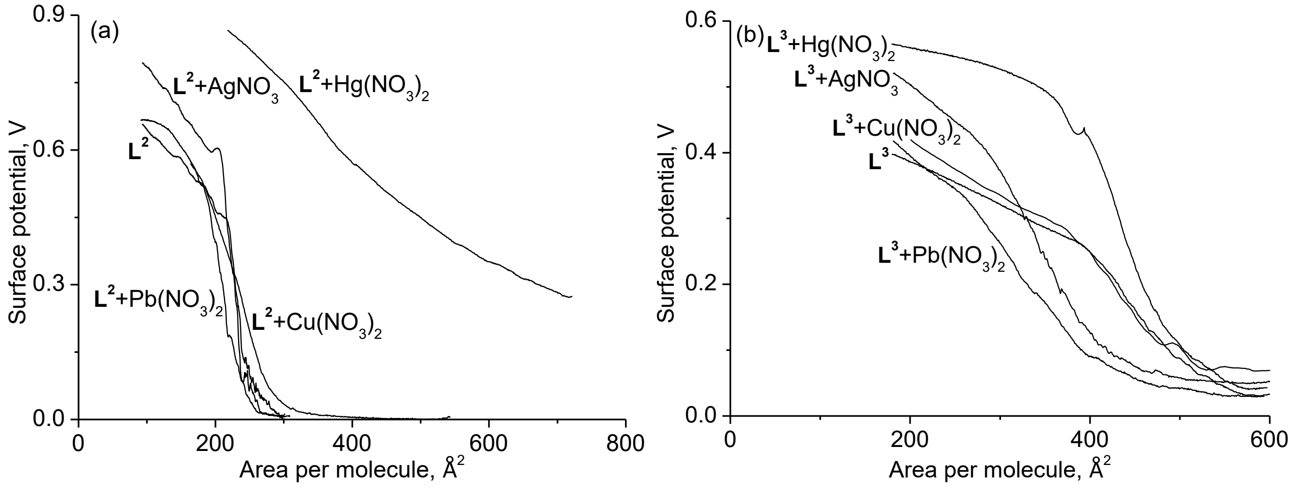

Figure S12. SPOT–*A* isotherms of ligands (a) **L²** and (b) **L³** ($c = 5 \times 10^{-5}$ *M* in $CHCl_3$) over deionized water or salt solution.

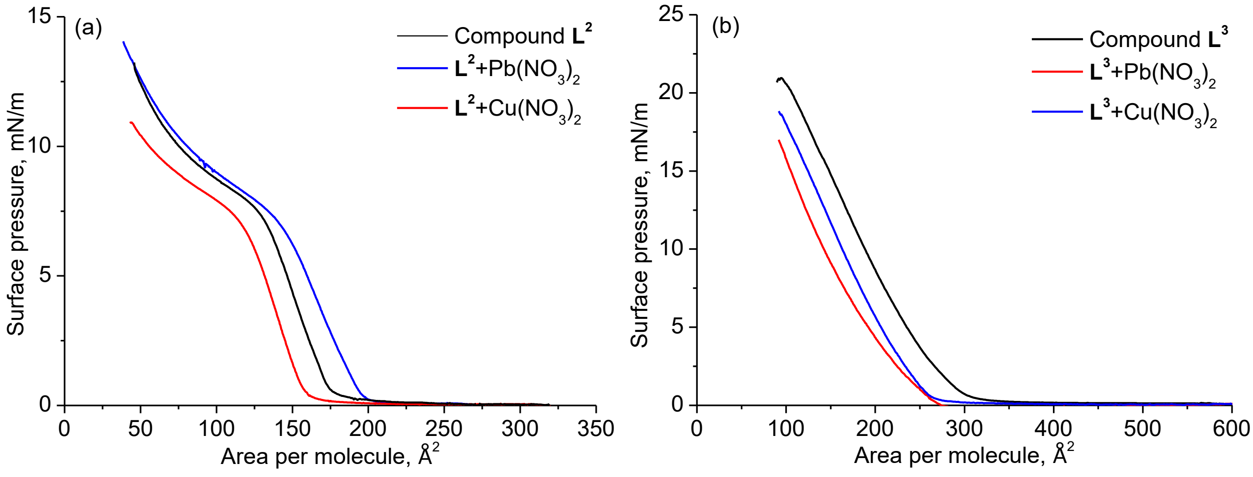

Figure S13. π–*A* isotherms of ligands **L²** and **L³** over deionized or salt water subphase ($c = 5 \times 10^{-5}$ *M* in $CHCl_3$).

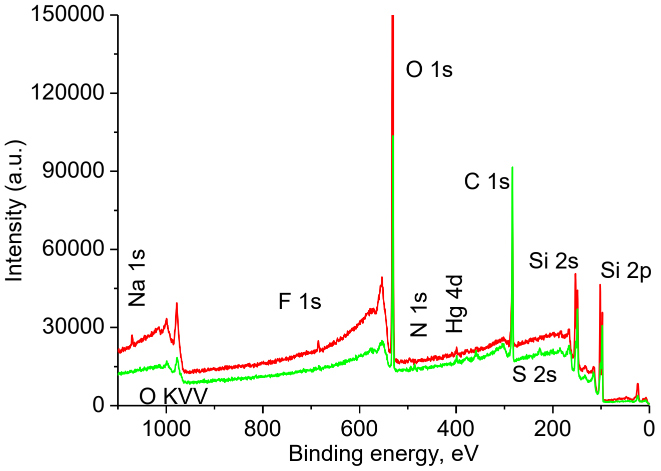

Table S3. Main characteristics of the components of XPS spectrum of Langmuir–Blodgett film-coated quartz

Name, state	Position, eV	Intensity, a.u.	FWHM, eV
$Si2p_{3/2}$, Si^0	99.34	8410	0.60
$Si2p_{1/2}$, Si^0	99.95	4205	0.50
Si^+	100.30	1657	0.84
$Hg4f_{7/2}$	101.16	2910	1.18
Si^{2+}	101.86	940	0.97
Si^{4+}	103.20	8173	1.52
$Hg4f_{5/2}$	105.42	2216	1.19

Figure S14. XPS spectra of bare quartz (red curve) and LB film on quartz formed by monolayer of compound $\mathbf{L^3}$ over 10^{-4} *M* $Hg(ClO_4)_2$ solution (green curve).

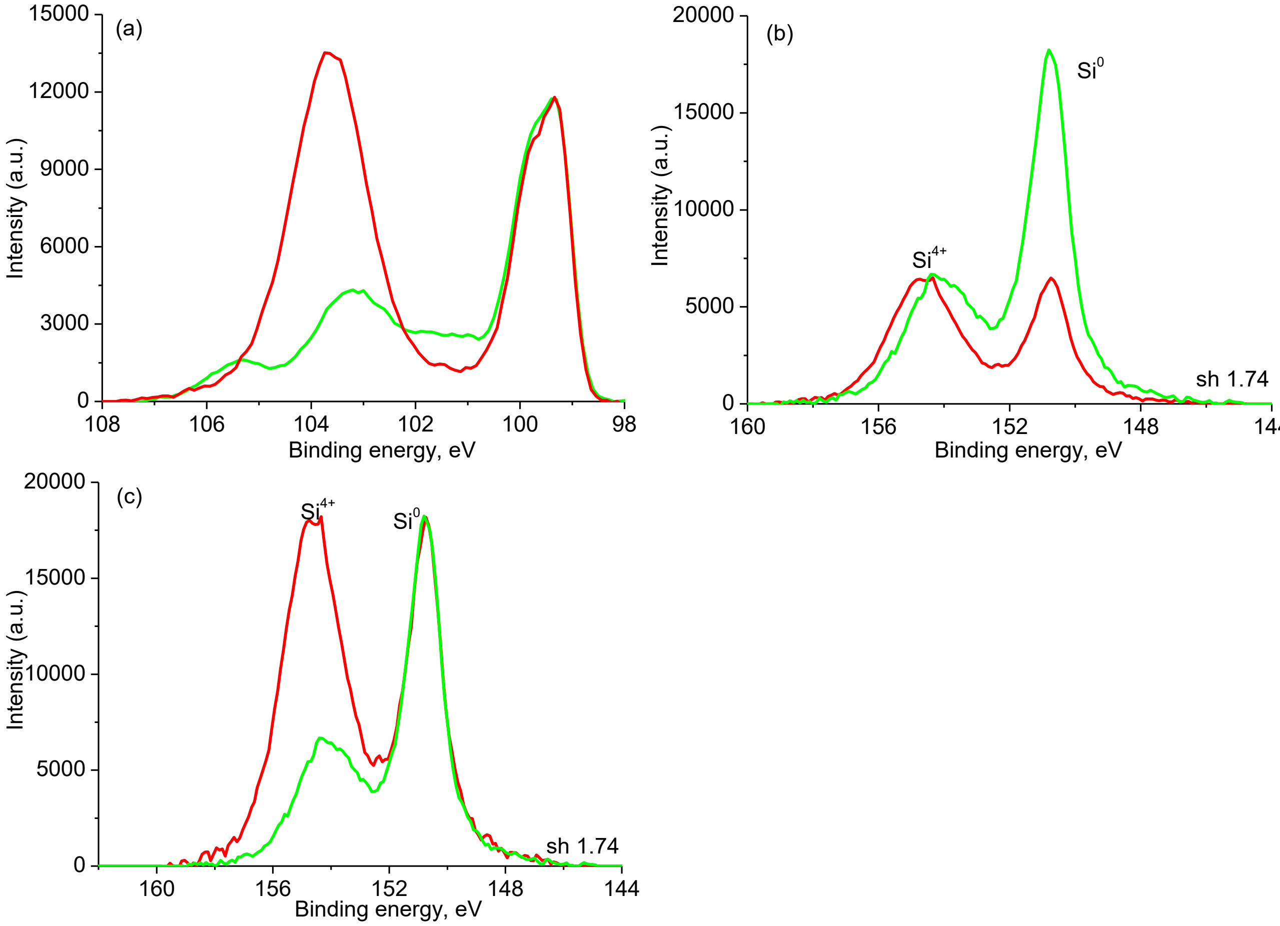

Figure S15. Partial XPS spectra of (a) Si2p and (b,c) Si2s regions of bare quartz (red curve) and LB film of compound $\mathbf{L^3}$ with $Hg(ClO_4)_2$ (green curve) normalized by the intensity of (a,c) low- and (b) high-energy peaks.
